# Development and evaluation of a clinical algorithm to monitor patients on antiretrovirals in resource-limited settings using adherence, clinical and CD4 cell count criteria

**DOI:** 10.1186/1758-2652-12-3

**Published:** 2009-03-04

**Authors:** David Meya, Lisa A Spacek, Hilda Tibenderana, Laurence John, Irene Namugga, Stephen Magero, Robin Dewar, Thomas C Quinn, Robert Colebunders, Andrew Kambugu, Steven J Reynolds

**Affiliations:** 1 Infectious Diseases Institute, Makerere University, Kampala, Uganda; 2 Johns Hopkins University School of Medicine, Baltimore, USA; 3 SAIC Frederick, MD, USA; 4 National Institute of Allergy and Infectious Diseases, National Institutes of Health, USA; 5 Institute of Tropical Medicine and University of Antwerp, Antwerp, Belgium

## Abstract

**Background:**

Routine viral load monitoring of patients on antiretroviral therapy (ART) is not affordable in most resource-limited settings.

**Methods:**

A cross-sectional study of 496 Ugandans established on ART was performed at the Infectious Diseases Institute, Kampala, Uganda. Adherence, clinical and laboratory parameters were assessed for their relationship with viral failure by multivariate logistic regression. A clinical algorithm using targeted viral load testing was constructed to identify patients for second-line ART. This algorithm was compared with the World Health Organization (WHO) guidelines, which use clinical and immunological criteria to identify failure in the absence of viral load testing.

**Results:**

Forty-nine (10%) had a viral load of >400 copies/mL and 39 (8%) had a viral load of >1000 copies/mL. An algorithm combining adherence failure (interruption >2 days) and CD4 failure (30% fall from peak) had a sensitivity of 67% for a viral load of >1000 copies/mL, a specificity of 82%, and identified 22% of patients for viral load testing. Sensitivity of the WHO-based algorithm was 31%, specificity was 87%, and would result in 14% of those with viral suppression (<400 copies/mL) being switched inappropriately to second-line ART.

**Conclusion:**

Algorithms using adherence, clinical and CD4 criteria may better allocate viral load testing, reduce the number of patients continued on failing ART, and limit the development of resistance.

## Introduction

The vast majority of Africans treated with antiretroviral therapy (ART) are not monitored with viral load testing. This is due to the cost and complexity of providing a reliable quantitative HIV RNA viral load service in resource-limited settings (RLS) [1,2]. It is therefore possible that a significant proportion of patients will suffer viral failure while continuing to take first-line ART [3]. This may encourage the development and accumulation of drug resistance [4-6].

A number of alternative measures of viral load for use in RLS are being investigated [1]. These include: direct measures of viral load, including HIV p24 based assays [7]; reverse transcriptase based assays [8]; filter paper transfer of whole blood or plasma for distant site bulk RNA quantification [9]; and qualitative dipstick assays that determine whether the viral load is detectable [10]. A few studies have investigated whether non-viral load-based parameters may predict viral status, including immune activation assays [11], adherence, clinical events, CD4+ T lymphocyte count (CD4 cell count) change and World Health Organization (WHO) failure criteria [12-15].

In this study, we investigated the utility of a combination of adherence patterns, clinical events and CD4 cell count criteria to determine the viral status of Ugandans on ART. The goal was to determine if the criteria listed above could be used to minimize viral load testing and detect viral failure among patients on ART [16]. A clinical monitoring algorithm was designed to classify patients into groups of viral status, including "failure likely", "failure possible", and "failure unlikely". The performance of this monitoring algorithm was then compared to an algorithm based on the current 2006 WHO treatment guidelines without viral load testing, which is currently the standard of care in many RLS [17].

## Methods

### Study design

This was a cross-sectional study of 496 Ugandans established on NNRTI-based ART. We evaluated combinations of adherence, clinical and laboratory variables to determine viral failure.

### Study setting

This study was performed at the adult clinic of the Infectious Disease Institute (IDI), Mulago Hospital, Makerere University in Kampala, Uganda. The IDI is one of Uganda's largest HIV treatment centres with more than 10,000 active patients and more than 5000 patients currently on free ART [18]. The IDI is supported by a College of American Pathologists-certified laboratory and is able to perform CD4 cell counts and viral load testing on site.

### Study participants

Patients were screened and included in the study if they were HIV-1 positive, aged >18 years, established on first-line NNRTI-based ART for ≥ six months and did not have viral loads monitored as per routine clinic practice. Patients with acute illness were excluded from the study.

### Data collection and study variables

From February 2006 to June 2006, 500 patients were enrolled at a rate of approximately 10 patients per clinic day. Patients were randomly selected from the clinic reception using a list of random numbers.

The study doctor carried out a structured interview and chart review using a study questionnaire. The questionnaire included detailed questions about treatment history, adherence to ART, clinical events and changes in laboratory parameters, including CD4 cell count since the start of treatment. CD4 cell counts are routinely ordered at the IDI every six months, with additional measurements taken if judged necessary by the treating physician.

Adherence was measured by self report, using a modified Adult AIDS Clinical Trials Group adherence questionnaire validated in our setting [19,20]. Participants were asked to report adherence patterns in the three days prior to enrolment, four weeks prior to enrolment, and since the initiation of ART. A visual analogue scale, as well as a question on whether treatment had ever been interrupted for more than two days, was included to assess adherence in the four weeks prior to enrolment and since the initiation of ART [21].

A blood sample was then taken for a complete blood count (ACT diff2 – Beckman Coulter, California, USA), CD4cell count and percentage (FACScalibur – Becton Dickenson, New Jersey, USA), and viral load (Amplicor HIV-1 Monitor v1.5 – Roche, Switzerland). The lower limit of detection for viral load was 400 copies/mL. An additional plasma sample was stored for each patient. Participants found to have a viral load of >1000 copies/mL underwent a genotypic resistance test (Trugene HIV-1 Genotyping Kit, Visible Genetics – Bayer Diagnostics, Leverkusen, Germany).

Examined variables included: months on ART; history of antiretroviral regimen limited to dual or triple nucleoside reverse transcriptase inhibitor therapy; history of maternal single-dose nevirapine to prevent vertical transmission; history of ever paying for ART; missing any ART during the last 30 days of treatment; ever missing ART for more than two days, current weight less than baseline weight; HIV-related symptoms, including prurigo and onset or relapse of opportunistic infection (OI); CD4 cell count change from baseline; 30% fall in CD4 cell count from on-treatment peak value; and WHO immunologic failure criteria (fall of CD4 count to pre-therapy baseline or below, 50% fall from on-treatment peak value, and persistent CD4 cell count of <100 cells/mm^3^).

A new or recurrent OI was defined according to 2006 WHO guidelines [17] as a WHO Stage 4 event (plus any severe bacterial infections or pulmonary tuberculosis) occurring six months after initiation of ART.

### Statistical analysis

We used χ^2 ^and Fisher's exact tests to compare categorical data, and the Kruskal-Wallis test to compare continuous variables. P values of < 0.05 were considered statistically significant. Univariate and multivariate logistic regression analysis was used to model variables associated with viral failure (>400 copies/mL).

We constructed the multivariate model by entering variables that were significant in the univariate analysis. To address multicollinearity, we examined variables that were strongly correlated and chose the variable with the greatest magnitude of association with viral failure to include in the multivariate model.

Variables in the final model were: gender; age; months on ART; history of paying for ART; ever missed more than two days of ART; 30% fall from peak CD4 cell count; and new or recurrent OI. A monitoring algorithm was then constructed using those parameters significantly associated with viral failure by multivariate logistic regression analysis.

Finally, we compared the ability of the regression-based algorithm and an algorithm using the WHO clinical and immunological treatment failure criteria [17] to classify patients according to viral status. We calculated sensitivity, specificity, positive and negative predictive value to determine viral failure <1000 copies/mL. Data were analysed using SAS version 8.2 (Cary, NC, USA).

### Ethical approvals

Informed consent was obtained from all the participants. Ethical approval for this study was obtained from the National Council of Science and Technology (Uganda) and from the National Institute of Allergy & Infectious Diseases (USA).

## Results

### Participant characteristics

Five hundred participants were enrolled, of which 496 had completed questionnaires and viral load results. Median age was 38.4 years (IQR, 33.5 to 43.7 years), and 311 (62.7%) were women. Forty-nine (9.9%) patients had a detectable viral load (>400 copies/mL). Thirty-nine (7.9%) patients had a viral load of >1000 copies/mL. Detectable viral loads ranged from 416 to 447,000 copies/mL.

The median duration of ART was 13 months (IQR, 10 to 16 months). The median CD4 cell count at baseline, before starting ART, was 90 cells/mm^3 ^(IQR 35 to 156 cells/mm^3^). The median CD4 cell count gain on treatment was 138 cells/mm^3 ^(IQR, 76 to 224 cells/mm^3^).

Eleven participants developed a new or recurrent OI on ART. These included *Pneumocystis jiroveci *pneumonia (N = 2), cryptococcal meningitis (N = 3), pulmonary tuberculosis (N = 3), extrapulmonary tuberculosis (N = 1), Kaposi's sarcoma (N = 1), and severe bacterial infection (N = 2). One participant suffered episodes of both *Pneumocystis jiroveci *pneumonia and pulmonary tuberculosis. Of these 11, only three had viral failure, including two participants with cryptococcal meningitis and one participant with severe bacterial infection.

### Univariate and multivariate logistic regression analysis

Table 1 summarizes the univariate results for adherence patterns, clinical events and laboratory variables associated with viral failure. Odds ratio for self report of ART missed in the last 30 days was 1.9 (95% CI 0.9 to 4.1) and for ever missed more than two days of ART was 6.3 (95% CI 3.4 to 11.8).

**Table 1:** Univariate analysis of variables associated with viral failure in 496 Ugandans on ART at the Infectious Diseases Institute, Kampala, Uganda

Variable	Total	Undetectable viral load N = 447	Detectable viral load N = 49	Odds ratio	P-value
Sex					
Male	185 (37%)	171 (38%)	14 (29%)	0.6 (0.3–1.2)	0.18
Female	311 (63%)	276 (62%)	35 (71%)	Referent	

Age, median (yrs)	38.4	38.4	37.6	...	0.29*

Mos. on ART, median^2^	13.0	12.9	14.6	...	0.002*

Non-HAART ever					
Yes	8 (2%)	6 (1%)	2 (4%)	3.1 (0.6–15.9)	0.18**
No	488 (98%)	441 (98%)	47 (96%)	Referent	

Hx of maternal nevirapine					
Yes	7 (1%)	5 (1%)	2 (4%)	3.8 (0.7–19.9)	0.1
No	489 (99%)	442 (99%)	47 (96%)	Referent	

Selfpay for ART					
Yes	86 (17%)	68 (15%)	18 (37%)	3.2 (1.7–6.1)	0.0002
No	410 (83%)	379 (85%)	31 (63%)	Referent	

Missed ART in last 30 days					
Yes	62 (12%)	52 (12%)	10 (20%)	1.9 (0.9–4.1)	0.08
No	434 (88%)	395 (88%)	39 (80%)	Referent	

Ever missed >2 days					
Yes	78 (16%)	55 (12%)	23 (47%)	6.3 (3.4–11.8)	<0.001
No	418 (84%)	392 (88%)	26 (53%)	Referent	

OI, new or relapse^4^					
Yes	11 (2%)	8 (2%)	3 (6%)	3.6 (0.9–14.0)	0.08**
No	484 (98%)	438 (98%)	46 (94%)	Referent	

CD4 gain from baseline^1^	138 (N = 417)	138 (N = 380)	146 (N = 37)	...	0.45*

CD4 <100, persistent^4^					
Yes	39 (8%)	32 (7%)	7 (14%)	2.2 (0.9–5.2)	0.08
No	457 (92%)	415 (93%)	42 (86%)	Referent	

30% fall from max^					
Yes	39 (8%)	29 (6%)	10 (21%)	3.8 (1.7–8.4)	<0.001
No	456 (92%)	418 (94%)	38 (79%)	Referent	

50% fall from max^,^4^					
Yes	12 (2%)	10 (2%)	2 (4%)	1.9 (0.4–8.9)	0.32**
No	483 (98%)	437 (98%)	46 (96%)	Referent	

Current CD4 < base^1,4^					
Yes	25 (6%)	20 (5%)	5 (14%)	2.8 (1.0–8.0)	0.04
No	392 (94%)	360 (95%)	32 (86%)		

Any WHO CD4 criteria					
Yes	66 (13%)	55 (12%)	11 (22%)	2.1 (1.0–4.3)	0.047
No	430 (86%)	392 (88%)	38 (78%)		

Any WHO CD4/OI criteria					
Yes	74 (15%)	62 (14%)	12 (24%)	2.0 (1.0–4.1)	0.048
No	422 (85%)	385 (86%)	37 (76%)		

*Kruskal-Wallis; **Fisher's exact test, ^N = 495, ^1 ^Due to missing value of CD4 cell count at baseline, N = 417; ^2 ^Due to missing value, N = 492; ^3 ^Due to missing value, N = 350; ^4 ^WHO failure criteria

CD4 cell count was measured by change in CD4 cell count from baseline, 30% fall and 50% fall from maximum achieved, persistent CD4 cell count of <100 cells/mm3, and CD4 cell count at study visit below baseline. WHO criteria were evaluated by univariate analysis of immunologic (CD4 cell count-based) criteria (OR, 2.1; 95% CI 1.0 to 4.3) and immunologic criteria and Stage 4 disease (OR, 2.1; 95% 1.0 to 4.2).

In the multivariate logistic regression model, ever missing ART for more than two days (OR, 5.2; 95% CI, 2.5 to 11.0) and 30% fall from peak CD4 cell count (OR, 3.9; 95% CI, 1.6 to 9.4) were significantly associated with viral failure (>400 copies/mL) after adjustment for gender, age, months on ART, history of paying for ART, and new or recurrent OI. Due to missing data for months on ART (N = 492) and 30% fall from peak CD4 cell count (N = 495), the multivariate results are based on 491 participants.

### Monitoring algorithms

**Figure 1:**
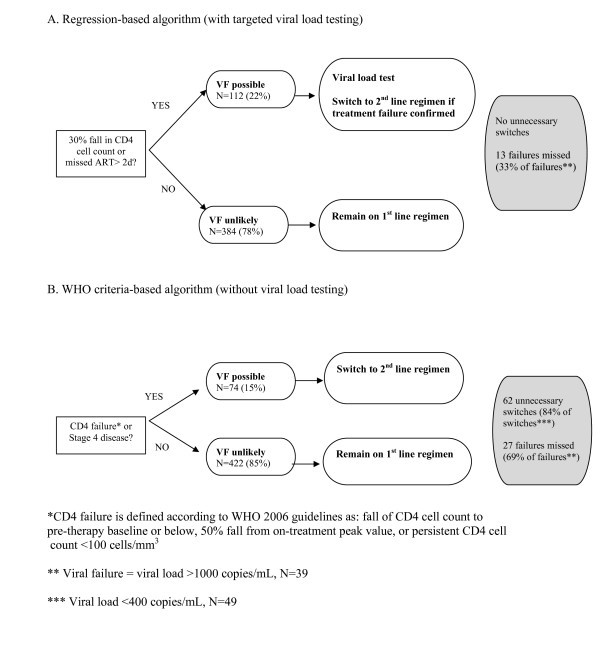
Two clinical algorithms to monitor for viral failure (VF) in 496 Ugandans on ART at the Infectious Diseases Institute in Kampala, Uganda.

The parameters significantly associated with viral failure (>1000 copies/mL) by multivariate logistic regression, ever missing ART for more than two days, and 30% fall in CD4 cell count were used to construct a monitoring algorithm (Figure 1a). Participants who met either criteria were classified as "failure possible" and were recommended for viral load testing (N = 112).

Those patients without either of these parameters were classified as "failure unlikely" and were not recommended for viral load testing (N = 384). According to the regression-based algorithm, no combination of parameters was predictive of viral failure. Therefore it was not possible to categorize participants as "failure likely" or recommend a second-line ART regimen without viral load testing.

In a WHO-based algorithm (Figure 1b), patients with immunologic failure and Stage 4 disease (not including lymph node TB, uncomplicated TB pleural disease, oesophageal candidiasis, and recurrent bacterial pneumonia occurring after six months of therapy) were recommended to switch to second-line ART without viral load testing (N = 74). Patients without these criteria were recommended to continue first-line ART (N = 422).

### Clinical utility of monitoring algorithms

The performance of the algorithms was assessed by sensitivity, specificity, and positive and negative predictive value, and then compared to an algorithm based on the WHO treatment failure criteria (Table 2). The regression-based algorithm identified patients with viral failure >1000 copies/mL with sensitivity of 67% and specificity of 82%, and identified 22% of patients for viral load testing. Thirty-three percent of patients with viral failure would continue first-line ART.

**Table 2:** Test performance characteristics of the regression-based and WHO-based monitoring algorithms to determine viral failure (>1000 copies/ml) in 496 Ugandans on ART at the Infectious Diseases Institute in Kampala, Uganda

	Sensitivity (95% CI)	Specificity (95% CI)	PPV (95% CI)	NPV (95% CI)	% Failures missed (1-sensitivity)	% Switched unnecessarily**	% Patients tested
Regression-based variables (30% CD4 fall or ever missed >2 days) with viral load testing [see Figure 1]	67% (63–71%)	100%	100%	97% (96–99%)	33%	0%	22%

Regression-based variables (30% CD4 fall or ever missed >2 days) without viral load testing	67% (63–71%)	82% (79–85%)	24% (20–28%)	97% (96–99%)	33%	18%	0%

WHO-based criteria (CD4 failure* or Stage 4 disease) without viral load testing [see Figure 1]	31% (27–35%)	87% (84–90%)	16% (13–19%)	94% (92–96%)	69%	14%	0%

WHO-based criteria (CD4 failure* or Stage 4 disease) with viral load testing	31% (27–35%)	100%	100%	94% (92–96%)	69%	0%	15%

*CD4 failure is defined according to WHO 2006 guidelines as: fall of CD4 cell count to pre-therapy baseline or below, 50% fall from on-treatment peak value, or persistent CD4 cell count <100 cells/mm^3^

**Viral load <400 copies/mL

Sensitivity of the WHO-based algorithm was 31% and specificity was 87%. This approach would not involve any viral load testing, would leave 69% of patients with viral failure on first-line ART, and would inappropriately switch 14% of those with viral suppression (<400 copies/mL) to second-line ART.

In a modification to the WHO-based algorithm, those patients meeting the criteria of CD4 failure or Stage 4 disease could have viral load testing rather than switching to second-line ART. Results were similar if viral load of either >400 or >10,000 copies/mL determined viral failure (data not shown).

### Drug resistance

Drug resistance testing was performed in 38 of the 39 participants with viral load of >1000 copies/mL (Table 3). In four participants, no mutations were identified, and in three participants, it was not possible to amplify the virus.

**Table 3:** Genotypic drug resistance test results of 39 study participants on ART with viral load >1000 copies/mL

Study ID	Current ART	Previous ART	Viral load (copies/mL)	Mutations in RT
33	EFV/3TC/AZT	-	190,343	K103N,M184V

34	NVP/3TC/D4T	-	50,626	G190A,M184V,D67N,K219Q

65	NVP/3TC/D4T	-	118,402	V108I,Y181C,M184V,T210W

71	NVP/3TC/D4T	-	141,470	Y181C,M184V

87	EFV/3TC/D4T		30,661	K103N,V108I,M184V,T215F

88	EFV/3TC/AZT		42,764	K103N,P225H,M184V

92	NVP/3TC/D4T		34,335	Y181C,M184V

107	NVP/3TC/D4T	-	66,838	Y181C,M184V

150	NVP/3TC/D4T	-	1,309	K103N,V108I,M184V

158	EFV/3TC/AZT	NVP/D4T	220,347	K103N,V108I,P225H,M184V,M41L,D67N,K70R,V75M,T215Y,K219Q

160	NVP/3TC/D4T	EFV	32,840	Y181C,M184V,T69N

212	NVP/3TC/D4T	-	10,627	G190A,M184V

216	EFV/3TC/AZT	-	15,161	M41L

240	NVP/3TC/D4T	-	229,960	K103N,M184V

247	NVP/3TC/D4T	-	2,611	Y181C,G190A,M184V

302	NVP/3TC/D4T	EFV/AZT	3,564	K103N,Y181CM184V

326	EFV/3TC/AZT	NVP/D4T	148,750	K103N,G190A,M184V,D67N,K70R,K219Q

348	NVP/3TC/D4T	-	18,596	Y181C,M184V,K65R*

353	NVP/3TC/D4T	-	1,614	Y181C,M184V

354	NVP/3TC/D4T		4,326	K103N,V108I,M184V,T215F

364	NVP/3TC/D4T	-	17,232	K103N,M184V

377	NVP/3TC/D4T	-	13,088	G190A,M184V

380	EFV/3TC/AZT	-	2,814	K103N,G190A,M184V

407	NVP/3TC/D4T	-	25,634	G190A,M184V

427	NVP/3TC/D4T	-	98,367	K103N,M184V,T215Y

459	NVP/3TC/D4T	-	13,404	G190S,M184V

463	NVP/3TC/D4T	-	54,432	Y181C,G190A,M184V

467	NVP/3TC/D4T	-	30,253	M184V,D67N,K70R,K219E

472	NVP/3TC/D4T	ABV	11,806	G190A,M184V,D67N,K70R,K219Q

477	NVP/3TC/D4T	-	39,783	V108I,Y181C,M184V,D67N,K70R,K219Q

487	NVP/3TC/D4T	EFV/AZT/TDF	17,980	K103N,Y188L,M184V,M41L,L210W,T215Y

*Previous use of tenofovir, abacavir, didanosine not elicited

KEY: NVP = nevirapine, EFV = efavirenz, 3TC = lamivudine, D4T = stavudine, AZT = zidovudine, TDF = tenofovir, ABC = abacavir

Significant mutations of the reverse transcriptase region were identified in 31 of the 35 (89%) participant samples in which amplification was successful [22]. All but one of these participants had mutations conferring resistance to either lamivudine (M184V) and/or NNRTIs (K103N, V108I, Y181C, G190A, P225H). Twelve participants (34%) had one or more thymidine analogue mutations (TAMs).

## Discussion

This study compares two different clinical algorithms to monitor patients on ART in a setting where access to viral load testing is limited. The optimal algorithm would have both high sensitivity and specificity for viral failure in order to minimize resistance, unnecessary switching from first-line regimens, and cost of viral load testing. However, the variables (adherence patterns, clinical events and CD4 cell count) are surrogates for viral load with less than perfect sensitivity and specificity.

We are concerned that patients may develop viral resistance due to continued exposure to a failing antiretroviral regimen. Therefore, we are interested in algorithms that screen for viral failure with high sensitivity. In this urban, public clinic-based population, the most sensitive algorithm to predict viral failure was based on parameters identified by multivariate regression (ever missing ART for more than two days, and 30% fall in CD4 cell count) with sensitivity of 67% and specificity of 82%.

This sensitivity of 67% represents a notable increase when compared to the 31% sensitivity of the WHO criteria. Potentially, using this algorithm with targeted viral load testing (of patients with either criterion) would minimize false positive results and reduce unnecessary switching to second-line agents, as would occur with the WHO-based algorithm if viral load testing was not used (see Figure 1) [12,23,24]. However, the sensitivity and specificity obtained with this regression-based algorithm may be different in other patient populations.

Also, the WHO treatment failure criteria were not designed to identify patients with early viral failure, but rather to facilitate decisions regarding switching patients to second-line ART in RLS. The WHO guidelines are used as a standard across many RLS. It is our view that this standard of care needs to be improved to reduce the late detection of viral failure and to minimize unnecessary switching of patients to second-line ART.

The regression-based analysis identified a history of ART interruption of more than two days as a significant risk for viral failure. Other studies have also found adherence history to be strongly associated with viral status [25-30].

While the best method for assessing adherence in busy African ART clinics has yet to be defined [20,30-32], careful assessment and support for 100% adherence is a very important and affordable tool in the optimization of viral response to ART. Recent poor adherence must be addressed before switching patients in RLS to more complicated and costly regimens.

A CD4 cell count fall of 30% was also associated with viral failure. In contrast, Bisson *et al *[13] and others [33,34], found that a gain in CD4 cell count was useful to detect viral suppression in patients on ART. We found no significant difference in CD4 lymphocyte count gain between those with and without viral failure, and CD4 cell count gain from baseline was not associated with viral outcome. We used a 30% fall from peak CD4 cell count to represent a significant change in CD4 cell count and to account for both laboratory and biological variation [35].

Of note, in this study, a 30% fall in the CD4 cell count was found to be more useful than the WHO recommended criterion of a 50% fall and was the only CD4 lymphocyte-related variable strongly associated with viral failure. Immunological poor responders (for example, persistent CD4 cell count of <100 cells/mm^3^) with undetectable viral loads were classified as unnecessary switches in this study. This is because there is no clear evidence to justify the additional cost and bill burden of switching these patients to a PI-based regimen in RLS [36].

Other parameters, including use of single-dose nevirapine, weight loss, or new or worsening OIs, were not associated with viral failure. This may be partly explained by the low prevalence of viral failure and the low OI rate in this study population. The majority of OIs occurred during the first six months of ART. Most episodes were not associated with viral failure and may have been related to the immune reconstitution inflammatory syndrome.

The inclusion of parameters that were not associated with viral failure, such as OIs and other CD4 criteria, did not improve the performance of the algorithm. In fact, we found no significant improvement in sensitivity, and specificity was reduced. Using these additional parameters would therefore require more viral load testing for little improvement in the number of viral failures detected.

By identifying patients with viral failure earlier, non-adherence can be addressed and resistance prevented. Furthermore, patients with resistance may be switched sooner to an effective second-line regimen to limit the evolution of resistance. The correct viral load cut-off for making this switch in RLS is unclear, especially when resistance testing data is rarely available [37]. We emphasised a cut-off of 1000 HIV RNA copies/mLas it is unlikely to be explained by viral "blips" [38]and allows an earlier diagnosis of viral failure [39,40].

The resistance data described in Table 3 shows that the majority (89%) of participants with a viral load of >1000 copies/mL have resistant virus. If patients with viral failure are allowed to continue on first-line ART, then it is likely that resistance mutations will accumulate [4-6] and reduce the effectiveness of second-line ART [41].

In this cohort, 34% of patients tested developed TAMs. Notably, the WHO guidelines recommend that patients continue first-line ART with detectable viral levels (<10,000 copies/mL) if the regimen is providing clinical benefit [17].

We are concerned that the current standard may lead to viral resistance and the need for more expensive ART regimens in the long term [42]. Given the lack of resistance testing in RLS, a modification to the proposed algorithm might be that patients identified with viral failure be re-tested after a period of intensive adherence support and only switched if they remain in viral failure. However, this would increase the cost of viral load testing [14].

The viral failure rate of 9.9% was unexpectedly low. While other cohort data from the IDI [29] and other African centres [3,43-46] have reported excellent 12-month outcomes, this result is likely to have been affected by survival bias. Due to the cross-sectional nature of this study, our results may not account for early losses to follow up (from deaths, etc.) and therefore provide an underestimate of the true viral failure rate. The cross-sectional design of our study also limits our method of adherence measurement and creates the possibility of recall bias.

Prospective studies using ongoing adherence measurements, including pharmacy refills, pill counts at monthly visits and other methods, would be subject to less recall bias and may provide a more accurate measure of adherence. The low number of viral failures and clinical events in this study limited its power to explore the relationship between a number of parameters and viral outcome. It is therefore important that the hypotheses explored here are investigated in larger multi-centre studies.

Finally, the results of this study were based upon a single viral load measurement. The diagnosis of viral failure ideally should be made after at least two measurements of viral load failure [47].

Adherence, CD4 cell count, and clinical criteria may identify those at risk for viral failure and better allocate viral load testing in RLS. Increased sensitivity of monitoring algorithms may reduce the number of patients continued on failing ART regimens and limit the development of viral resistance.

For this approach to improve care, however, ART providers must find extra funding for additional viral load testing [2,48]. Lower-cost, simple viral load testing methodologies are urgently needed for RLS to improve monitoring of patients on ART and to avoid widespread drug resistance.

## Footnote

This data was presented at the 14^th ^Conference on Retroviruses and Opportunistic Infections, held in Los Angeles, USA, from 25 to 28 February 2007 (abstract 531)

## Competing interests

The authors declare that they have no competing interests.

## Authors' contributions

DM, LJ, SJR, TCQ, RC and AK contributed to study design, study oversight and conduct and manuscript writing. LS and LJ performed data analysis and contributed to manuscript writing. RD performed laboratory analyses and contributed to manuscript writing. HT, SM and IN contributed to study conduct and final manuscript writing.
